# Linkage of national clinical datasets without patient identifiers using probabilistic methods.

**DOI:** 10.23889/ijpds.v7i3.2067

**Published:** 2022-08-25

**Authors:** Helen Blake, Linda Sharples, Katie Harron, Jan van der Meulen, Kate Walker

**Affiliations:** 1 London School of Hygiene and Tropical Medicine; 2 Royal College of Surgeons of England; 3 University College London (UCL) Great Ormond Street Institute of Child Health

## Objectives

To develop a step-by-step process for probabilistic linkage of national clinical and administrative datasets without personal information, providing guidance on selecting variables for linkage, estimating match weights, and choosing the probabilistic linkage threshold. To validate this process against deterministic linkage using patient identifiers.

## Approach

We undertook probabilistic linkage without personal information using electronic health records from the National Bowel Cancer Audit (NBOCA) and Hospital Episode Statistics (HES) databases for bowel cancer patients undergoing emergency surgery in England. We selected linkage variables based on completeness, and ability to discriminate between matches and non-matches, assessed using a novel score derived from m-probabilities and u-probabilities. Taking deterministic linkage using patient identifiers as the reference-standard, we calculated sensitivity and specificity of probabilistic linkage, plotted a Receiver Operating Characteristic curve across alternative thresholds of match weights, and compared patient characteristics and estimates from fitted regression models between linkage methods.

## Results

When considering the ability to discriminate between matches and non-matches, patient and administrative variables tended to discriminate better than clinical variables. 81.4% of NBOCA records were linked to HES using probabilistic linkage, versus 82.8% using deterministic linkage. Most NBOCA records were linked to HES using both methods (8,427/10,566). Probabilistic linkage had over 96% sensitivity and 90% specificity compared to deterministic linkage using patient identifiers. Patients that linked deterministically, but not probabilistically, were younger and more likely to have emergency admission, but otherwise had similar characteristics. Regression models for mortality and length of hospital stay according to patient and tumour characteristics were not sensitive to the linkage approach.

## Conclusion/Implications

Probabilistic linkage without personal information can be used as an alternative to deterministic linkage using patient identifiers, or as a method for enhancing deterministic linkage. It allows analysts outside highly secure data environments to undertake linkage while minimising costs and delays, protecting data security, and maintaining linkage quality.

